# Fractured Stents: The Silent Trigger of a Popliteal Artery Aneurysm

**DOI:** 10.1155/crvm/4628882

**Published:** 2025-10-03

**Authors:** R. Teh, M. Garbowski

**Affiliations:** ^1^Department of Vascular Surgery, St John of God Subiaco Hospital, Subiaco, Western Australia, Australia; ^2^Department of Vascular and Endovascular Surgery, Sir Charles Gairdner Hospital, Nedlands, Western Australia, Australia; ^3^Faculty of Medicine and Health Sciences, The University of Western Australia, Crawley, Western Australia, Australia; ^4^Medical School, Curtin University, Bentley, Western Australia, Australia

**Keywords:** endovascular complications, interventional radiology, vascular surgery

## Abstract

Popliteal artery aneurysms (PAAs) are largely attributed to arteriosclerotic disease processes, with a rare aetiology of infective and traumatic origin. This disease may be complicated by acute limb ischaemia, which could result in limb loss. Therefore, early management of symptomatic aneurysms, or asymptomatic aneurysms > 2 cm, is suggested. We present a unique case of PAA secondary to a fractured femoropopliteal stent and discuss ongoing challenges toward the management of femoropopliteal disease, along with treatments for PAA.

## 1. Introduction

Popliteal artery aneurysms (PAAs) comprise 85% of aneurysms involving the peripheral arterial system [1], with up to 50% presenting with significant complications of acute limb ischaemia (ALI). The disease is characterised by an artery of diameter 1.5 times normal, generally at > 15mm [2], with male preponderance [3]. The development of PAAs has been largely attributed to arteriosclerotic lesions leading to turbulent flow, with dilation of the artery beyond the adductor hiatus into the popliteal fossa [4]. Other causes include mycotic and syphilitic aneurysms, as well as traumatic aneurysms [5, 6].

Due to the risks of thrombosis, embolism and rupture, elective repair of PAAs confers better outcomes. A palpable mass or increased pulsatility at the popliteal fossa could prompt a diagnosis, with duplex ultrasound being the most useful radiological test [1]. Additional investigations could be achieved with computed tomography (CT), magnetic resonance imaging (MRI) or conventional arteriography [1]. Aneurysm expansion rates increase with increasing aneurysm size [1], and repair is recommended for symptomatic aneurysms, asymptomatic aneurysms over 2 cm or aneurysms with significant mural thrombus [3].

Surgical treatment aims to exclude the aneurysm, preventing distal embolization and providing effective revascularization [7]. Approaches can be broadly classified into open or endovascular modalities, with studies showing improved 1-year primary patency with open repair. Yet, endovascular approaches have fewer wound complications and a shorter length of stay, with a limb salvage rate of 96.5% [7, 8]. Complications of endovascular interventions include early thrombosis and stent occlusions, stent stenosis, stent migration, endoleaks, continuous sac expansion and stent fractures [9]. There is particular concern for the deployment of a stent across a knee joint, with repetitive stress from bending increasing the risk of kinking, fractures and stent occlusion [9]. For similar reasons, stent fractures are a concern when managing femoropopliteal disease.

We present a case of PAA, secondary to a distal Type IV fractured drug-coated, self-expanding Eluvia (Boston Scientific, Marlborough, Massachusetts, United States) stent in the femoropopliteal artery (FPA) segment, managed with an endovascular approach. Consent was obtained from the patient for publication.

## 2. Case Presentation

A male in his early 70s was under surveillance for a small abdominal aortic aneurysm (AAA) of 3.5 cm and a left-sided PAA of 17 mm. His vascular history is significant for a right-sided femoral to popliteal vein bypass graft for lower limb ulceration, as well as three overlapping left-sided FPA stents for claudication. His other past medical history is significant for ischaemic heart disease (IHD) and atrial fibrillation (AF), with previous coronary artery bypass grafts (CABGs), cardiac ablation and left atrial appendage occlusion (LAAO).

**Figure 1:**
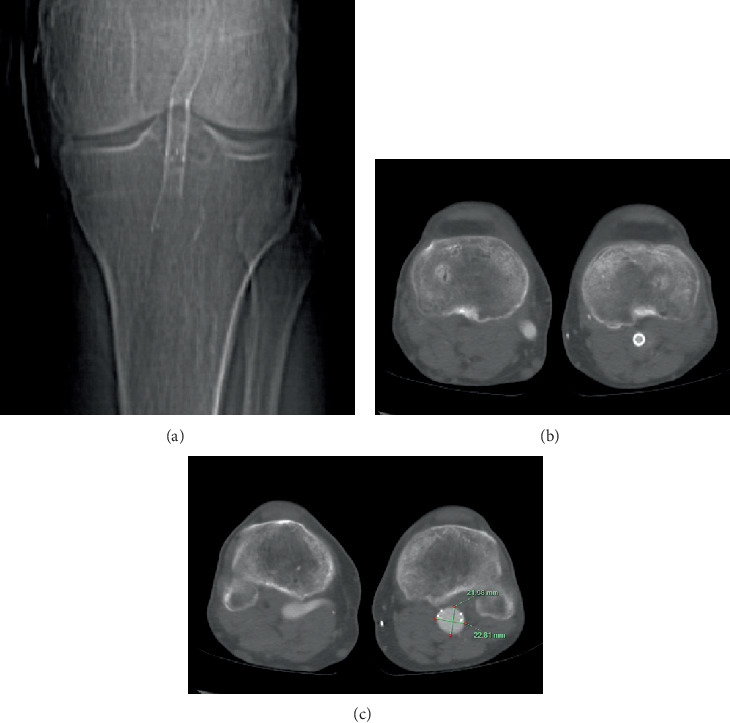
Computed tomography angiogram. Scout image demonstrating (a) fractured stent, (b) with the superficial artery stent patent just above the level of the popliteal artery aneurysm and (c) subsequent aneurysmal dilation of approximately 23 × 22 mm.

At subsequent 12-month follow-up, duplex ultrasound demonstrated stable AAA size; however, there was an increase in PAA size to 22 mm. This was confirmed with a CT angiogram demonstrating a 23 × 22 × 20 mm PAA reported to arise immediately distal to his FPA stents ending 18 mm above the origin of the anterior tibial artery (Figure 1). He was experiencing short-distance claudication in his left calf on walking, along with intermittent episodes of sharp pain.

**Figure 2:**
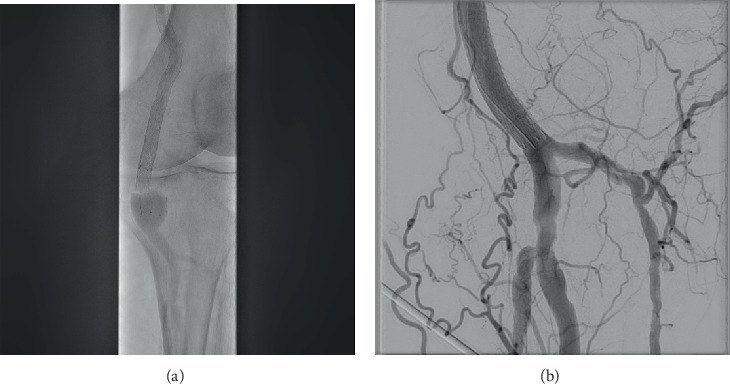
Intraoperative digital subtraction angiography. (a) Left distal popliteal artery aneurysm with Type IV fracture of the distal segment of Eluvia stent demonstrated within the aneurysmal sac. (b) A Viabahn stent was deployed with good exclusion of the aneurysmal sac.

Given the increasing sac size, an elective procedure was planned for aneurysm exclusion with a covered stent. Arterial access was obtained through the right common femoral artery (CFA) via retrograde puncture, with an up-and-over approach to gain access to the left SFA, secured using a 7 French 90 cm Destination Peripheral Guiding Sheath (Terumo). A diagnostic angiogram demonstrated a Type IV fracture of the Eluvia stent, with the fractured segment located within the PAA (Figure 2). The aneurysm was crossed with a 0.018⁣^″^ V-18 guidewire (Boston Scientific), repaired with a 7 × 100-mm covered self-expandable Viabahn stent (W.L. Gore & Associates, Flagstaff, Arizona, United States). A final angiogram run demonstrated a fully excluded aneurysm with preserved three-vessel run-off at the tibial arteries. Manual pressure was used for the access site puncture.

There were no complications from the procedure. The patient was kept overnight for monitoring and discharged home, restarting on his regular daily dose of direct oral anticoagulant (DOAC) and 100 mg aspirin on discharge. At follow-up, clinical improvement was achieved.

## 3. Discussion

We present a unique case of PAA secondary to a fractured stent. Although fractured stents of the FPA segment have been reported to occur in 2%–65% of implanted stents [10], aneurysm formation from stent fractures is rarely reported in the literature. Lee et al. [11] reported on a 71-year-old male with a fractured self-expanding bare metal stent in the SFA, complicated first by in-stent occlusion, which was recanalized, and subsequent Rutherford 3 ALI with aneurysmal changes and in-stent occlusion, managed with a covered stent. Manola et al. [12] reported on a coronary artery aneurysm secondary to a stent fracture, with distal in-stent restenosis presenting with inferolateral electrocardiographic (ECG) changes. These published cases of stent fractures are complicated with in-stent restenosis distal to the fractured site; however, this was not demonstrated in our case.

Silveira et al. [13] observed in a cohort of 39 patients with 55 FPA stents a critical period of 2 years for the occurrence of device fractures. In detecting fractured stents, Pang et al. [14] compared CT angiography, conventional cine-angiography and intravascular ultrasound (IVUS) in their ability to detect coronary artery stent fractures. CT angiography was found to have the highest sensitivity (80.7%) and specificity (100%) [14]. There are no studies looking at the utility of duplex ultrasound in detecting stent fractures. Unfortunately, despite undergoing CT angiography, features of a fractured stent were not clear in this case, and findings were made during a diagnostic angiogram, stressing the importance of considering this differential with aneurysm development around a stent.

Management of PAA has traditionally involved ligation and restoration of arterial continuity with a vein bypass graft [15]. Endovascular PAA repair has conferred great advantages in avoiding major incisions and comorbidities associated with limb dissection, postoperative scarring and reducing hospital length of stay [15]. Yet, the popliteal artery does present as a hinge point in knee flexion, where biomechanical stresses can occur [15]. Other considerations include the suitability of access vessels, the availability of healthy proximal and distal segments for stent landing and fixation, along with the discrepancy of diameters of landing zones [15]. A randomised-controlled trial of open versus endovascular repair for asymptomatic PAA demonstrated that even though 1-year primary patency is better in open repair (100% vs. 86.7%), secondary patency for endovascular repair was 100% at 12 and 36 months [7]. The Viabahn stent graft (W.L. Gore & Associates Inc., Flagstaff, Arizona, United States) is a flexible, relatively kink-resistant, polytetrafluoroethylene (PTFE) graft, recommended as an option for use in the popliteal artery [15].

Greater influence of external forces, compression, torsion and elongation also makes treatment of FPA lesions challenging. A prospective study of 239 patients who underwent elective endovascular therapy, in a total of 333 affected limbs, for FPA lesions with nitinol stents reported a 14% incidence of stent fracture. Higher rates of fractures were seen in more severe lesions as per TransAtlantic Inter-Society Consensus (TASC) classification and longer lesion length [16]. The authors found that 1-year primary patency was worse with fractured stents (68%) compared to unfractured stents (83%); however, the difference between these two groups became insignificant after 2 years. Specifically, Type II multistrut fractures were found to have poor outcomes of early primary patency, whereas Types I and III fractures did not have significant effects [16]. Types IV and V fractures were not present in the study. Scheinert et al. [17] demonstrated a 13.2% risk of fracture in FPA stents < 8 cm in length, 42.4% in stents > 8–16 cm and 52.0% for stents measuring > 16 cm.

Drug-coated balloons (DCB), drug-eluting stents (DES) and covered stents reduce neointimal hyperplasia and progression of atheromatous disease that influence primary patency [18]. In addition, DES and covered stents also prevent immediate elastic recoil and intimal dissection [18]. Results from the SIROCCO, STRIDES and ZILVER-PTX studies support the use of drug-eluting self-expandable stents, with superior primary patency compared to bare metal stents in the FPA [18]. In addition, Viabahn covered stents were also found to have better primary patency rates compared to conventional balloon angioplasty [19] and bare metal stents [20]. Yet, DCBs remain an effective strategy for FPA disease to avoid risks of restenosis whilst avoiding stent fracture [21]. The THUNDER and femoral paclitaxel (FemPac) trial both demonstrated the superiority of paclitaxel-coated balloons compared to noncoated, with lower restenosis rates in the femoropopliteal segment as well as target-lesion revascularization [21]. More recently, the ILLUMENATE trial demonstrated the efficacy of a rapid-release drug delivery mechanism to infuse paclitaxel into FPA lesions with 12-month primary patency of 87%. Other delivered drugs currently trialled include dexamethasone in the DANCE (dexamethasone infusion to the adventitia to enhance clinical efficacy after femoropopliteal revascularization) trial [21]. Subadventitial dexamethasone administration conferred improved ankle–brachial index up to 12 months and comparable 6-month patency rates to DES.

Emerging technology for the FPA segment includes bioabsorbable scaffolds, which are temporary stents that achieve the desired immediate result whilst disappearing over time [18, 21]. These scaffolds, made of bioabsorbable poly-L-lactic acid or metal alloys such as magnesium, could also have antiproliferative drug elution [21]. The REMEDY study, a multicentre nonrandomised registry of a biodegradable stent for the SFA, showed 94.6% immediate technical success, 70.9% 6-month primary patency and 21.9% target lesion revascularization rates [21]. The GAIA study had binary restenosis of 67.9% with 1-year reintervention rates of 57.1% [18]. Although these results are promising, there are no good randomised controlled studies currently available to demonstrate efficacy.

In summary, we present a rare incidence of an aneurysm secondary to a Type IV fracture to a DES of the FPA segment. We propose that clinicians need to be aware of this possibility, especially within the first 2 years of stent deployment. Successful exclusion of the aneurysm along with the fractured stent segment was achieved with a Viabahn covered stent, which is recommended in the FPA given its flexible profile. However, we present current literature exploring alternatives such as DCBs and emerging devices to avoid complications of stent fractures or restenosis. Biodegradable stents may be a promising area in the treatment of diseases of the femoropopliteal segment; however, further studies are still required to determine their efficacy.

## Data Availability

The data that support the findings of this study are available on request from the corresponding author. The data are not publicly available due to privacy or ethical restrictions.
